# Supplementary material for: Earthquake slip surfaces identified by biomarker thermal maturity within the 2011 Tohoku-Oki earthquake fault zone
Source: Nat Commun. 2020 Jan 27;11:533. doi: 10.1038/s41467-020-14447-1 (PMC6985169; doi:10.1038/s41467-020-14447-1)
Supplement: Supplementary file 1 — Supplementary Information [file 41467_2020_14447_MOESM1_ESM.pdf]

Supplementary Materials for

Earthquake slip surfaces identified by biomarker thermal maturity within the 2011 Tohoku-Oki Earthquake fault zone

by Rabinowitz et al.

Supplementary Notes

Supplementary Note 1: JFAST Stratigraphy

The lithostratigraphy of the JFAST core was described in the JFAST Science Party Report[1] and refined through a chemostratigraphic analysis[2]. Here, we summarize the stratigraphy presented by Rabinowitz et al. (2015), which provides the most detailed correlations between JFAST samples and their protolith in the reference core used in this study (Site 436). The shallowest sediments recovered at JFAST are Unit A2 Late Miocene mudstones. Below these, at a depth of ~690 mbsf, lie Holocene to Late Pliocene Unit A1 diatomaceous silty mudstones. This age inversion suggests a fault between the two units, though core recovery in this interval is insufficient to analyze variations in the thermal maturity of the faulted material. The Unit A1 material persists to a depth of ~820 mbsf where a <5 m thick layer of Early Miocene Unit C2 pelagic clay, interpreted as the main slip zone of the Tohoku-Oki earthquake[3–5], was recovered. Thrust into this pelagic clay layer is a sliver of Unit A3 mudstone. Below the pelagic clay layer is an inverted sequence of Unit B underlain by Unit A3 and then A2. This series of age inversions implies the presence of faults at ~824, 825, and 832 mbsf. The deepest sediments in the JFAST core are Unit D partially silicified clay. Here, the 60 Myr age gap is interpreted as another fault at a depth of ~833 mbsf[2].

Supplementary Note 2: Biomarkers at JFAST

Alkenone concentrations (C_{37} total) measured in the JFAST core (Supplementary Fig. 1a) demonstrate relatively constant values in the top part of the core while concentrations drop and are more variable closer to the plate boundary (at depths ≥ 810 mbsf). The alkenone concentration at Site 436 changes over 3 orders of magnitude, with the oldest samples having the lowest alkenone concentration. The oldest sedimentary units (Units C and D) have alkenone concentrations that are below the detection limit. This trend in alkenone concentration is likely dominated by changes in productivity, rather than changes in preservation of alkenone molecules. The low concentrations of middle-late Miocene samples reflect low productivity as the site passed under oligotrophic subtropical gyre waters. Increasing concentrations occurred in the late Miocene as the site entered the more productive western boundary current. Although the

changes in concentrations of alkenones throughout Site 436 are large, the concentrations in JFAST samples are only compared to the alkenone concentrations for the corresponding sedimentary unit at Site 436 (Supplementary Fig. 1b).

The alkenone unsaturation index ($U^{k'}_{37}$) generally decreases at Site 436 from higher values during the middle Miocene to lower values in the late Miocene (with a few exceptions). Interpreted as sea surface temperature, this trend indicates cooling from middle Miocene to present times as seen in many other regions[6]. Unlike other biomarker proxies used in this study, $U^{k'}_{37}$ values increase with increasing thermal maturity[7]. We find that $U^{k'}_{37}$ values in Site 436 and the shallower sediments in the JFAST core are similar (if somewhat variable, Supplementary Fig. 2b), whereas samples from the bottom of the JFAST core near the plate boundary (Supplementary Fig. 2a) are consistently higher, with four unambiguous thermal anomalies (Fig. 2b).

In order to address concerns that local heterogeneities in deposition of organic material and dilution by inorganic sediments (dust, volcanics, terrigenous sediment, carbonate etc.) might yield variable alkenone concentrations, we analyzed the TOC of samples from JFAST and Site 436 (Supplementary Fig. 3). TOC values at JFAST are constant for most of the Unit A1 sediments and within ~0.0008 g/g from the range of Site 436 Unit A1 values. The exception to this is at ~818 mbsf, where the TOC values in Unit A1 JFAST samples drop and are within ~0.0015 g g^{-1} from the minimum values observed in Site 436. The other sedimentary units show similar consistency in TOC with Unit A2 samples at JFAST within 0.0007 g g^{-1} from Site 436 values, Unit A3 and B samples at JFAST within 0.0003 g g^{-1} from Site 436 values, and Unit C samples at JFAST within 0.0002 g g^{-1} from Site 436 values. When the ratio of alkenones to TOC in the JFAST core is considered (Supplementary Fig. 3c), the major alkenone anomalies remain, indicating that the alkenone anomalies that we observe are larger than any variations caused by differential dilution between JFAST and Site 436.

CPI at Site 436 shows no clear trend with age and values fluctuate around a CPI of ~3. Units A1 and A3 have a larger range of CPI values than the other sedimentary units, with Unit B showing the least variability (Supplementary Fig. 4b). CPI values in the hanging wall sediments in the JFAST core are relatively constant, with values near the upper end of the range of values for Unit A1 measured in Site 436 (Supplementary

Fig. 4a). While significantly lower CPI values are observed near the plate boundary, the variance in the initial CPI values as measured at Site 436 limits the number of samples that exhibit anomalous values with respect to the whole range of initial CPI (Fig. 2c). Anomalies with respect to the range of CPI values in the corresponding sedimentary unit at Site 436 are observed at 822 and 824 mbsf.

ADI is fairly constant throughout Site 436, with most values lying between an ADI of ~1 and 1.5 (Supplementary Fig. 5b). This stability in ADI is also seen in the hanging wall sediments in the JFAST core with lower ADI values approaching the plate boundary (Supplementary Fig. 5a). Again, an anomaly with respect to the range of possible initial values is seen at 822 mbsf in the pelagic clay. The agreement of both *n*-alkane parameters that a biomarker anomaly exists here supports the conclusion that this is a localized seismic structure within the pelagic clay. However, two other samples analyzed within the previously interpreted décollement layer (including a sample from the mudstone biscuit thrust into this layer) do not show clear evidence for fault heating (Fig. 2). This observation supports previous suggestions that some of the deformation in this layer has been accommodated through aseismic or distributed slip[8–10]. Another ADI anomaly is observed at 832 mbsf (Supplementary Fig. 5a), supporting the interpretation of a thermal anomaly implied by the alkenone anomalies.

The presence of biomarker anomalies in samples near to sections of the core that are affected by drilling disturbance raises the question of whether these anomalies could result from so-called drill-bit metamorphism (DBM). This effect has been observed in the oil industry due to shear heating along the drill-rock interface. However, DBM has been shown to be a significant problem in cores drilled with polycrystalline-diamond compact bits which allow for turbo-drilling (~1000 rpm) as opposed to cores drilled with conventional rotary drilling (~300 rpm)[11]. The rotation speed (fast versus slow) and mode of failure (shearing versus crushing) differ between high-speed and conventional drilling, and both high rotation and shearing contribute to DBM. The JFAST core was drilled

using conventional rotary drilling and, therefore, DBM is not expected to play a significant role here.

Supplementary Note 3: Analysis of minimum size earthquakes at damage structures in the JFAST core

We run a forward model for each sample that exhibits a biomarker anomaly (i.e. samples indicated in red in Fig. 2) to determine potential temperature rise from large earthquakes. As stated above, temperature rise is a function of shear stress and slip during the earthquake, as well as fault parameters such as fault thickness and rock properties. Parameters used to model temperature rise are taken from observations of the JFAST borehole and sediments (Supplementary Fig. 6), along with measurements of material properties from the core[12]. Shear stress on the fault is 0.54 MPa, as determined by JFAST borehole temperature decay measurements reported by Fulton et al. (2013). To analyze the potential for seismic slip, we use a sliding velocity of 1 m/s. Estimates of shallow displacement during the Tohoku-Oki earthquake range from ~40–80 m[13]. Our models span this range, and in addition, we explore displacements from 5–150 m to determine the minimum required slip magnitude to replicate the observed biomarker anomalies, with models conducted every 10 m displacement. Sediment density ρ=1850 kg m^{-3}, thermal diffusivity α=3.92x10^{-7} m^{2}s^{-1} and heat capacity c=1515.7 J kg^{-1}K^{-1} are based on measurements of samples recovered at JFAST[1,12]. We note that contact asperity-scale flash heating could lead to locally higher temperature-rise. However, most of the samples that we model are not taken from within a slip zone, making this effect unlikely to influence the observed signal. Additionally, the sample volume is much larger than these asperity-scale heat sources, so the observed signal would average the hot asperities with the surrounding sample.

We use core observations to find potential fault structures close to each anomalous biomarker sample. In some cases, samples were taken directly from a fault structure (samples PP829 and PP948), while other samples were from intact sections of the core. In those cases, we measure the distance to the closest potential fault and compare our thermal maturity to an equivalent off-fault distance in our model (e.g. Supplementary Fig. 8). We note that there is inherent uncertainty in these distance

measurements due to incomplete core recovery, which leads to uncertainty in spatial relationships between samples and fault structures. Missing material could contain faults or increase the true distance between a sample and the closest fault.

Active slipping thickness can be difficult to ascertain from structure data alone because there are often multiple localized slip zones within a fault and determining whether these represent separate events or if the whole fault was active during a particular earthquake is impossible. For samples associated with a clear structure with easily definable boundaries, the maximum thickness of the slipping zone is considered to be the thickness of that structure. To further constrain fault width, we establish a minimum and maximum possible thickness based on known temperature limits of two reactions. The biomarkers used in this study do not react at short timescales (minutes–days) below 120 ºC[7]. Faults with half widths greater than a maximum value (e.g. a_{max} = 4.8 cm assuming a displacement of 50 m; Supplementary Fig. 7a) do not generate enough heating to reach 120 ºC and therefore would not produce any measurable reaction. Furthermore, forward models indicate that a Tohoku-Oki-sized earthquake would not generate temperature above 120 ºC at distances more than ~5 cm from the fault, which is an important constraint for off-fault anomalies (Supplementary Fig. 7b). On the hotter end of the spectrum, we expect smectite clay to amorphize at 900 ºC[14,15]. Amorphous clay material has not been reported in the JFAST core, suggesting temperatures remained below 900 ºC for all samples. This provides a lower limit on the fault half-widths because of the larger temperature rise associated with thinner half-widths (e.g. temperature rise is above 900 °C for a_{min} = 0.26 cm and 50 m displacement; Supplementary Fig. 7a). By placing the modeled events along faults of different thicknesses, we get a range of possible temperature rises (e.g. Supplementary Fig. 9). We note that due to computational limitations, we restrict the minimum half-width to values ≥0.5 mm, a constraint which only affects models with slip less than 50 m. Below this half width value, for earthquakes with >50 m displacement, heat generation and heat diffusion balance out and peak temperature no longer increases for thinner a (Supplementary Fig. 7).

In addition to the possible range in fault half-widths, we also consider the potential cumulative biomarker thermal maturity from multiple slip events to explore the possibility that the fault experienced multiple earthquakes (e.g. Fulton and Harris, 2012).

In the case of multiple slip events, we limit the total slip on a fault to 3.2 km, based on the palinspastic reconstruction of Chester et al. (2013). This gives an end-member situation that all displacement accommodated in this plate boundary fault zone occurred on one of the faults observed at the JFAST site and gives a maximum number of events of any given magnitude for each structure[2,8,17]. Clearly, this approximation overpredicts the maximum number of earthquakes on each structure as no structure analyzed here or present in unrecovered sections of the JFAST core could have accommodated all 3.2 km of displacement.

As described in the Methods, these constraints are used to generate a series of probability distributions for different a, $N_{earthquake}$ combinations for a given slip distance for each sample (e.g. Supplementary Fig. 9 for 50 m of slip). The probability of each slip distance is then evaluated to determine whether earthquakes of that size could be hosted on the structure (Supplementary Fig. 10). For example, the 50 m symbols on Supplementary Fig. 10 are the maximum values from the probability distribution for each fault in Supplementary Fig. 9.

We are also interested in the smallest earthquake that could cumulatively generate the biomarker signal, given the constraint of 3.2 km total slip. This is a minimum constraint as it is the lowest slip magnitude where some of the models match the data within its uncertainty and that of the biomarker kinetics. Larger events could also have generated the observed biomarker signature on these faults as evidenced by the non-zero probabilities (fraction of models matching the data) as slip magnitude continues to increase (Supplementary Fig. 10).

The shallowest biomarker anomalies in the plate-boundary region were observed at ~818 mbsf in three samples between 817.4–817.9 mbsf (PP944, PP945, and PP727). While these samples are relatively closely spaced, they are far enough apart that it is unlikely that they all were heated by slip on the same fault (Supplementary Fig. 7b). Candidate structural features in Core 15 are observed at 817.5 mbsf, 817.6 mbsf, and ~817.8 mbsf (Supplementary Fig. 6).

While the structure at 817.6 mbsf appears more significant, it is too distant (>10 cm) to cause any biomarker anomaly at sample PP944 (817.485 mbsf). Even with multiple slip events, there would be no biomarker anomaly from this feature recorded at

this sample. One candidate structure near to PP944 is a high angle crack in the core, which is, indeed, close enough to have caused significant biomarker alteration; however, this feature appears very minor (Supplementary Fig. 6) and is unlikely to have accommodated 50–80 m of coseismic slip, much less to have hosted multiple megathrust earthquakes. Another nearby structure is a near horizontal gouge layer at 817.5 mbsf with a maximum thickness of ~1.5 cm (Supplementary Fig. 6), lying 0–4 cm away from PP944. Such horizontal features have been previously interpreted as resulting from drilling damage to the core rather than being tectonic structures[17,18], however due to its proximity to the reacted sample and the shallow dip of many of the larger features in the core, we consider this the most likely candidate for slip if the anomaly was generated during a megathrust earthquake. The thermal anomaly at this sample is best fit by a model with multiple earthquakes with a slip magnitude of ~40 m. The smallest event that could have contributed to the signal is a 10 m slip earthquake (Supplementary Fig. 10a, Supplementary Table 1). However, this result suggests that this fault would be one of the most significant within the core. Because of the lack of extensive damage and gouge generation around these features as well as the lack of any stratigraphic age reversals in this depth range[2], this outcome seems unlikely. Because the structures immediately adjacent this particular sample are small and thin, we also model a smaller slip magnitude with a higher shear stress than was measured for the Tohoku-Oki earthquake[12,19]. Slip during six smaller (1 m) events with a higher friction coefficient of μ=0.5 can also fit the biomarker data.

The next sample down, PP945, is adjacent to the ≤ 2 cm thick structural feature at 817.6 mbsf (0–3 cm away). This structure is at the border of a zone of fragmented core near the bottom of core 15R–1W that could be related to fault damage. The biomarker anomalies here are well fit by 30 m slip events and can be fit with a minimum size event of 10 m slip (Supplementary Fig. 10b).

Similarly, PP727 lies within damaged material recovered in 15R–CCW and near (0.5–10 cm) to a more highly comminuted region at 817.8 mbsf with a maximum thickness of ~9 cm (Supplementary Fig. 6). We note that the original thickness of this feature is likely not preserved due to drilling disturbance. In this case, the maximum thickness of the modeled slipping zone is limited by the minimum temperature of

biomarker reaction (Supplementary Fig. 7a). While this sample shows biomarker anomalies in alkenone concentration, $U^{k'}_{37}$, and ADI, the lack of an observed anomaly in CPI provides a strong additional constraint in our models. The anomalies observed in this sample are best fit by several 100 m slip events, implying that this sample could represent the cumulative effect of several large megathrust events. The minimum slip magnitude that could contribute to the biomarker anomalies measured in this sample is 30 m (Supplementary Fig. 10c, Supplementary Table 1). A relatively large amount of displacement here is consistent with the broad damage zone observed at this depth in the core (Supplementary Fig. 6).

We sampled three locations within the pelagic clay layer: at 821.8 (mudstone biscuit), 822.12, and 822.55 mbsf. Though the entire layer has been interpreted as having hosted displacement, with multiple features near the top of the recovered pelagic clay interpreted as localization features for seismic slip[8,9], only one of the three samples that we analyzed within this layer exhibited unambiguous biomarker anomalies. This indicates that, while the whole pelagic clay layer is significantly sheared, only certain locations have experienced localized seismic slip. We note that the pelagic clay is not expected to have any alkenones in it, based on the lack of alkenones in the Site 436 pelagic clay units (Supplementary Fig. 1b), and thus, the lack of alkenones in the pelagic clay samples cannot be interpreted in terms of biomarker thermal maturity. However, both the CPI and ADI show anomalies for sample PP829 (822.55 mbsf). This sample is within a region of structures interpreted as shear localization bands, which are ~0.5–1 cm thick and thus, distance from the fault is taken to be 0–10 cm (Supplementary Fig. 6). The *n*-alkane anomalies are well fit by a slip magnitude of 70 m (Supplementary Fig. 10d). Larger slip magnitudes are precluded because they would require a minimum half-width that is larger than that allowed by the observed thickness range of the shear localization bands. The biomarker anomalies in this sample require earthquakes with slip magnitudes of at least 10 m (Supplementary Fig. 10d, Supplementary Table 1).

Core 18 exhibits a significant amount of apparent damage[17,18] with few obvious localization features (Supplementary Fig. 6). This damage has been interpreted as a combination of damage induced by core recovery and tectonic damage related to the damage zone focused around the pelagic clay layer[18]. There is a stratigraphic inversion

above this core between the pelagic clay unit and the underlying mudstone unit[2] and both observations together imply a major fault below the pelagic clay layer. Sample PP730 lies within a significantly brecciated section of the core. While the brecciation in this core is extensive, a zone of elevated damage is about 4 cm thick (Supplementary Fig. 6). The sample was taken from ~4 cm away from this highly comminuted band, but could be closer (~1 cm) to a slip zone if the entire damage zone is considered. This sample is well fit by 40 m slip events and requires earthquakes with a minimum slip magnitude of 30 m (Supplementary Fig. 10e, Supplementary Table 1).

The biomarker anomaly observed at 825.6 mbsf in sample PP948 is found in the core catcher section of Core 18. This sample is found in a heavily brecciated section of the core, right above a stratigraphic inversion interpreted to be a fault by Rabinowitz et al. (2015), and is likely to be immediately adjacent to (or even within) the fault (Supplementary Fig. 6). The implication that this sample represents a seismic fault from structural observation is supported by the large amount of alkenone reaction (near complete alkenone destruction), though the lower reaction rates from the *n*-alkane parameters seem to have generated negligible reaction. Because alkenone concentration is below the quantification limit, constraints on the fraction of alkenones reacted are poor, though the chromatograms qualitatively appear to have lower alkenone concentrations than even the lowest alkenone concentrations observed in the Unit B samples at Site 436. The inability to accurately quantify alkenone concentrations in this sample also prevents us from determining the $U^{k'}_{37}$ value. Due to the relatively poor constraints on the level of biomarker reaction in this sample, we do not model the number of earthquakes that could be hosted on this fault and instead represent the sample with open red symbols in Figs. 2 and 3.

We find that two samples near the mudstone/silicifying clay boundary at ~833 mbsf[2] exhibit biomarker anomalies. The shallower sample (PP951, 832.515 mbsf) shows anomalies in both alkenone parameters but in neither of the *n*-alkane parameters. This sample is close to a peak in tectonic damage features[18], implying the presence of a fault at this depth (Supplementary Fig. 6). The biomarker anomalies measured in this sample are best fit by earthquakes of slip magnitude equal to ~90 m and can be fit by earthquakes with a minimum slip magnitude of 40 m (Supplementary Fig. 10f,

Supplementary Table 1). However, similar to sample PP944 described above, there are closer, smaller structures which could also explain the temperature anomaly with very small displacement on thin structures, and higher friction (Supplementary Fig. 6). The biomarker alteration in this sample can be fit by a model with two 1 m earthquakes and a higher friction coefficient of μ=0.5. The deeper sample, PP952, is constrained through structural observations of the JFAST core (Supplementary Fig. 6). This sample was taken ~1.5 cm away from a very visible contact between late Miocene mudstone and Cretaceous silicifying clay at 832.85 mbsf. This sample is well fit by 100 m slip events and also requires events with a minimum slip magnitude of 40 m (Supplementary Fig. 10g, Supplementary Table 1).

Supplementary Note 4: Temperature rise estimates and implications for dynamic weakening mechanisms

One of the central goals of earthquake mechanics research is to understand the dynamic weakening mechanisms that control seismic slip. This better understanding is essential for improving seismic hazard estimates because the conditions required for dynamic instability likely exert a strong control on where and when an earthquake will nucleate and how far it will propagate. A key parameter to constrain in the search for plausible dynamic weakening mechanisms is the coseismic temperature because many of these mechanisms are thought to be thermally activated[20].

We present a temperature constraint based on the minimum slip magnitude required to reproduce the biomarker anomalies in each sample. Specifically, we report the minimum temperature required for this minimum slip magnitude earthquake (Supplementary Table 1). Because this is a lower-bound limit for the temperature rise on these faults, plausible dynamic weakening mechanisms must be activated by temperatures greater than or equal to these temperature estimates. The average minimum temperature determined in this way is ~279±43 (1σ) ºC with a range of 233–335 ºC (Supplementary Table 1). We emphasize that this minimum temperature estimate is based on the minimum size earthquake that could replicate the observed biomarker anomalies in the allowable displacement. Because it is unlikely that one of the observed faults accommodated all, or even most, of the displacement in this décollement, the faults likely

hosted earthquakes with displacements higher than the minimum values reported here. Accordingly, the temperature experienced during these earthquakes could be significantly higher than these minimum temperatures. An upper bound on the temperature achieved in these faults comes from the fact that there have been no published observations of clay amorphization or pseudotachylyte to date. As discussed above, this limits the peak temperature to 900 ºC. However, any amorphous material in the JFAST core would likely be present only on very thin features and it is possible that finer-scale sampling in the future will reveal amorphous material. We emphasize that, even if such features are observed, this does not change the major finding of this paper: multiple faults in the JFAST core could have hosted megathrust earthquakes such as the Tohoku-Oki earthquake. We note that temperature estimates on natural faults are best constrained by the application of a wide range of paleoseismic indicators with different temperature sensitivities.

Supplementary Figures

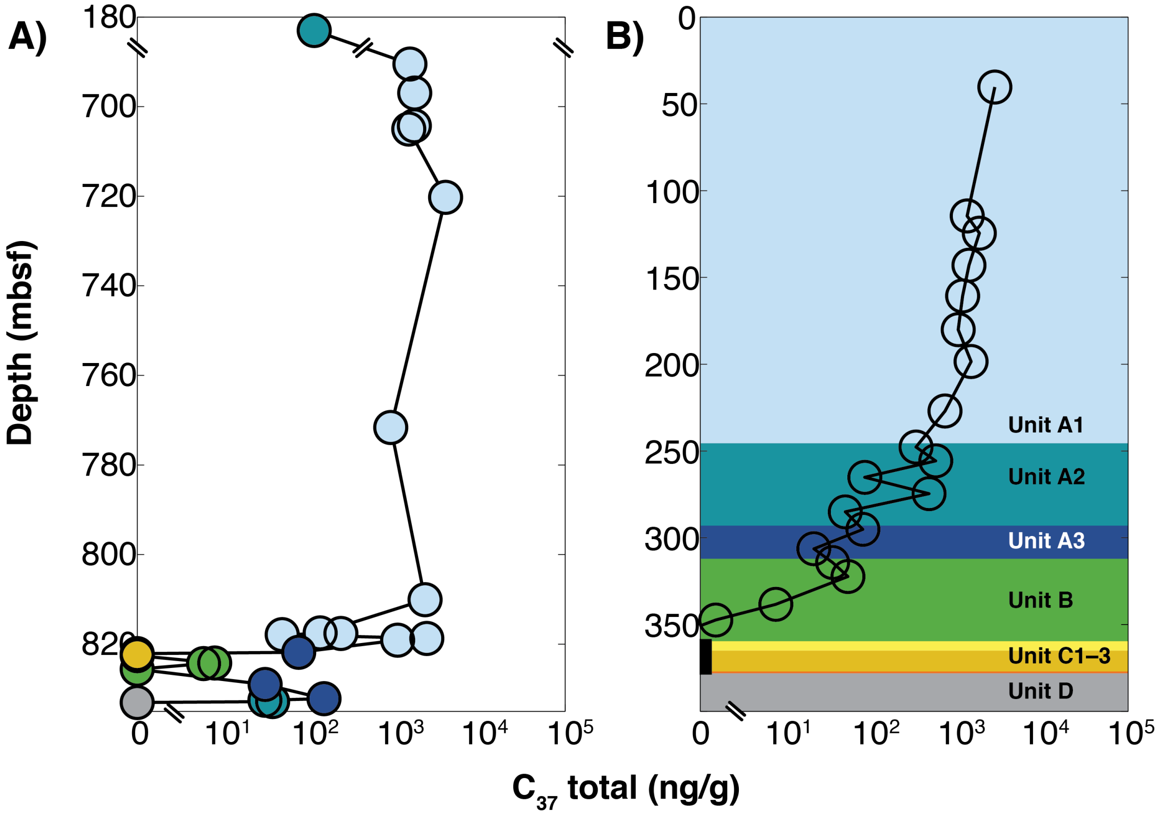

Supplementary Fig. 1. Alkenone concentrations at JFAST and Site 436

Alkenone concentrations measured in the JFAST core (A) and in the reference core, Site 436 (B). Colors indicate unit designations, labeled in (B), of the samples[2]. JFAST alkenone concentrations are constant in the shallower sections of the core while concentrations are decreased and more variable near the bottom of the core. In Site 436, concentrations decrease with depth and are below the detection limit in Unit C, represented by the black bar at these depths.

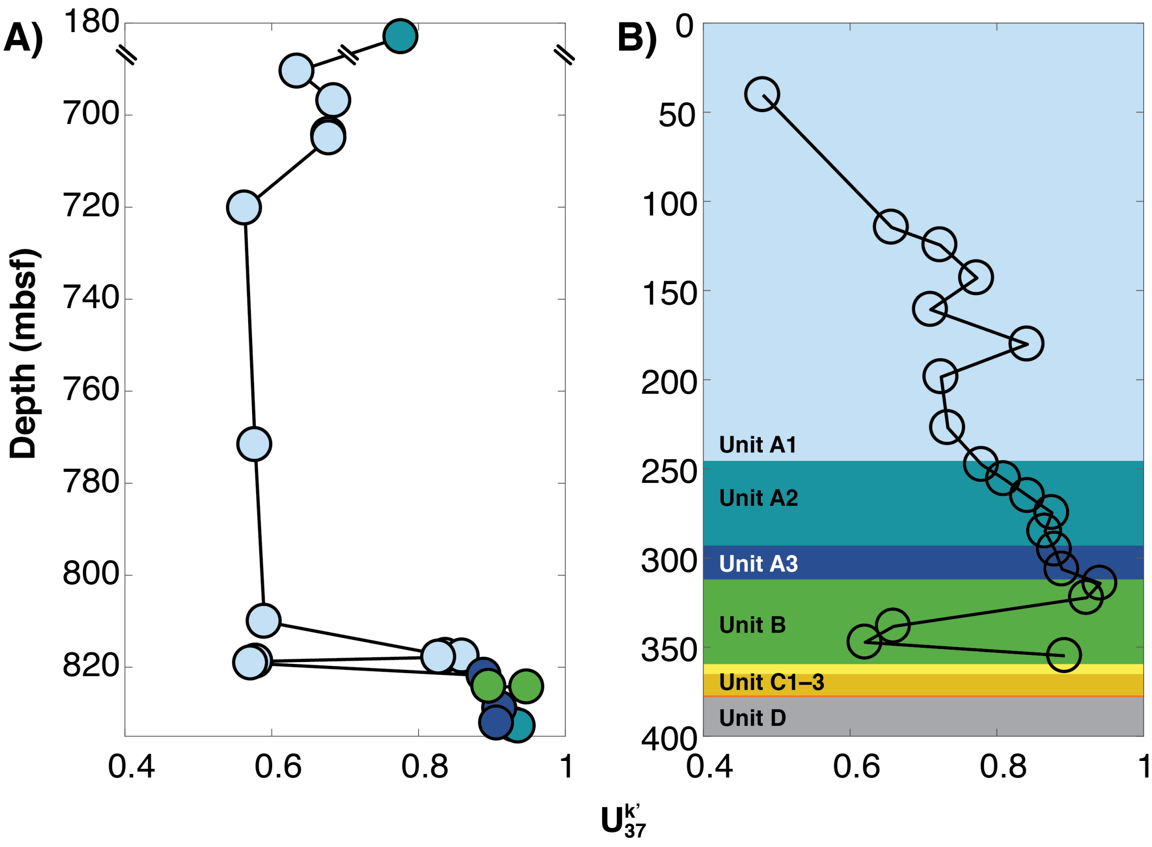

Supplementary Fig. 2. $U^{k'}_{37}$ at JFAST and Site 436

$U^{k'}_{37}$ measured in the JFAST core (A) and in the reference core, Site 436 (B). Colors correspond to sedimentary units defined by Rabinowitz et al. (2015). $U^{k'}_{37}$ values are constant near the top of the JFAST core, and near to the lower values observed in corresponding sedimentary units at Site 436. $U^{k'}_{37}$ values increase approaching the plate boundary. Note that $U^{k'}_{37}$ cannot be calculated in Unit C samples due to the lack of alkenones measured in these subunits.

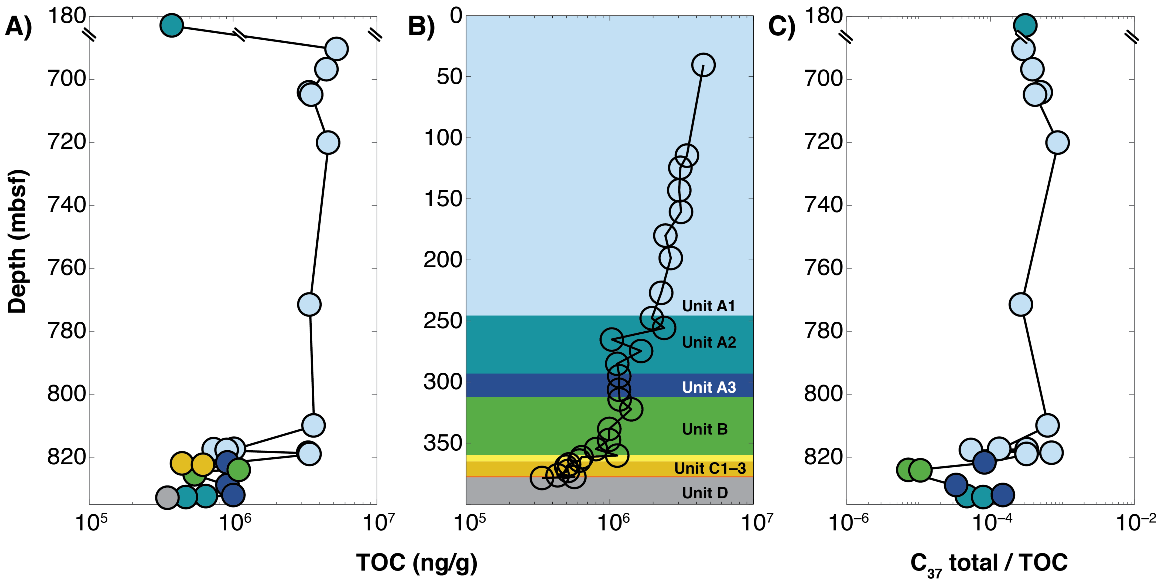

Supplementary Fig. 3. Total organic carbon at JFAST and Site 436

Total organic carbon (TOC) measured in the JFAST core (A) and in the reference core, Site 436 (B). The alkenone/TOC ratio is also shown for the JFAST core (C). While some variability in TOC is observed in samples near the plate boundary in the JFAST core, anomalies can still be seen in the alkenone/TOC ratios, implying that alkenone anomalies are beyond that which can be explained by depositional effects.

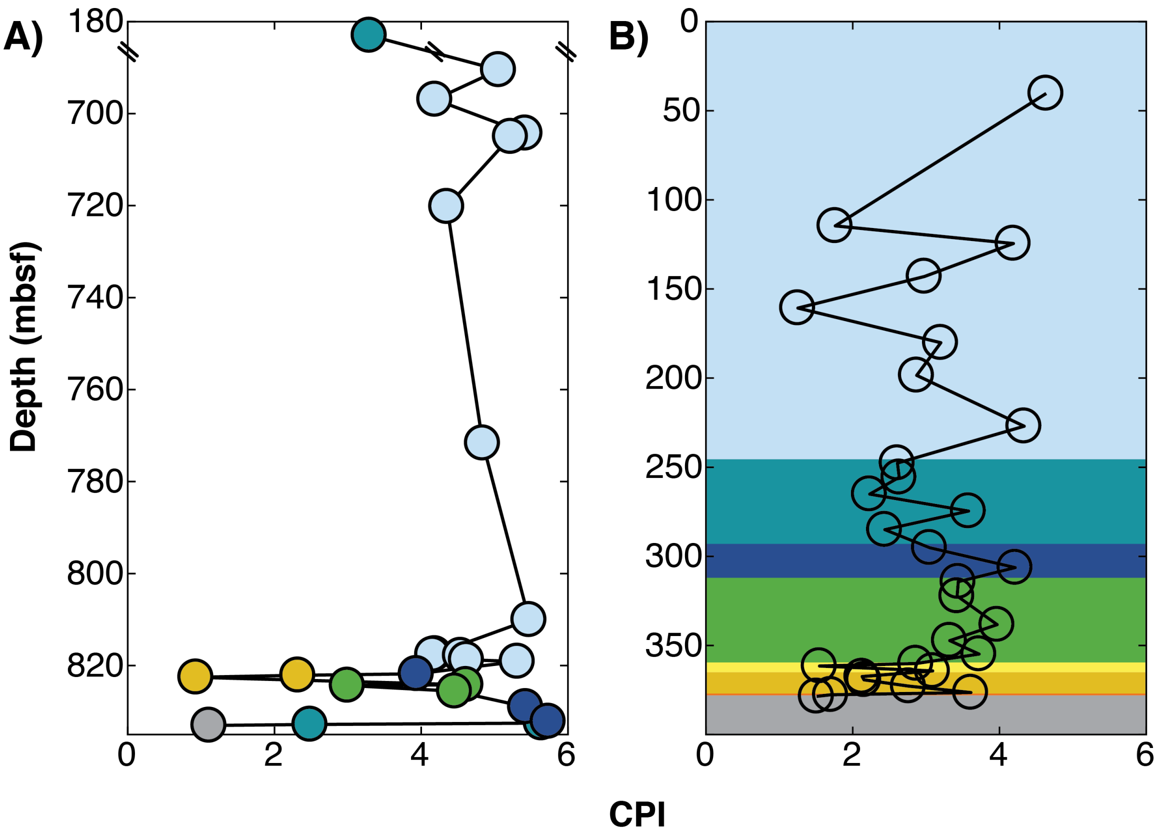

Supplementary Fig. 4. Carbon Preference Index (CPI) at JFAST and Site 436

CPI values measured in the JFAST core (A) and in the reference core, Site 436 (B). CPI at the top of the JFAST core is within the range of observations for corresponding units at Site 436, while values in decrease below those observed in corresponding units at Site 436 in two samples approaching the plate boundary region.

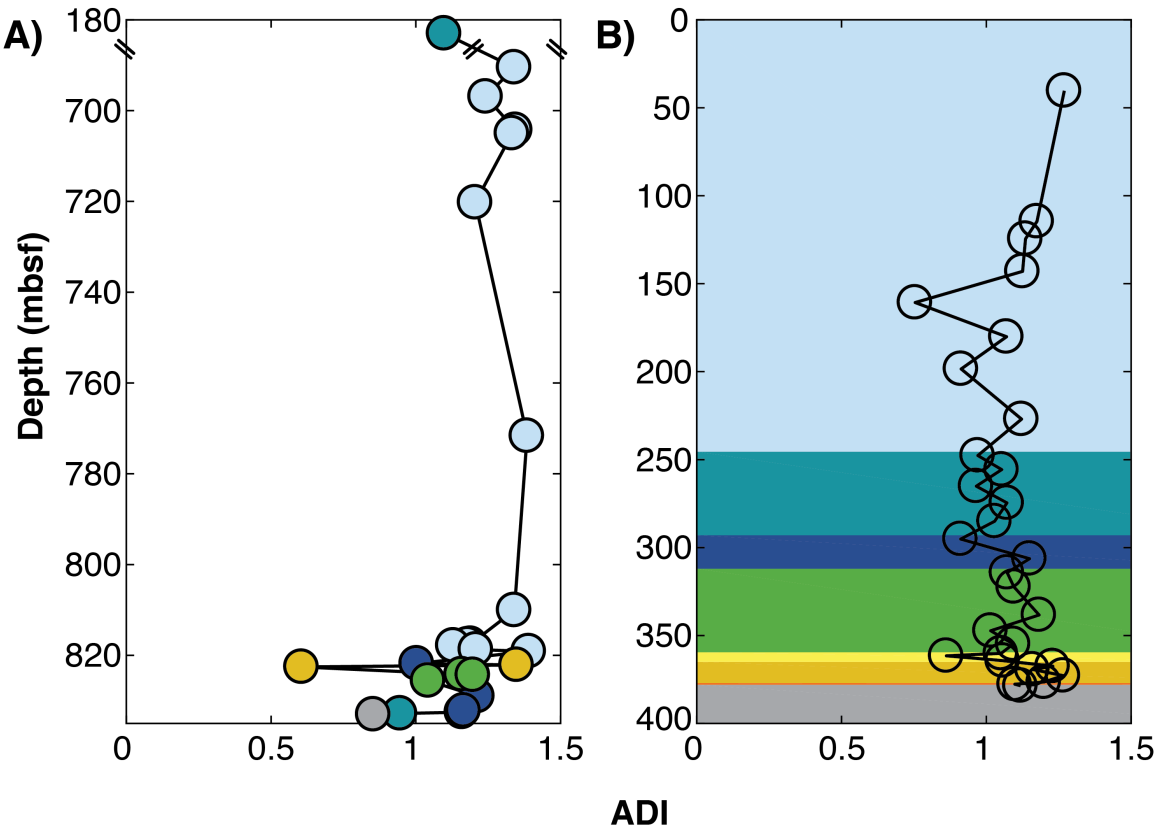

Supplementary Fig. 5. Alkane Distribution Index (ADI) at JFAST and Site 436

ADI values measured in the JFAST core (A) and in the reference core, Site 436 (B). ADI values in the JFAST core are constant in the top portion of the core and show more variability and decreased values (beyond those observed at Site 436) in the deeper samples near the plate boundary region.

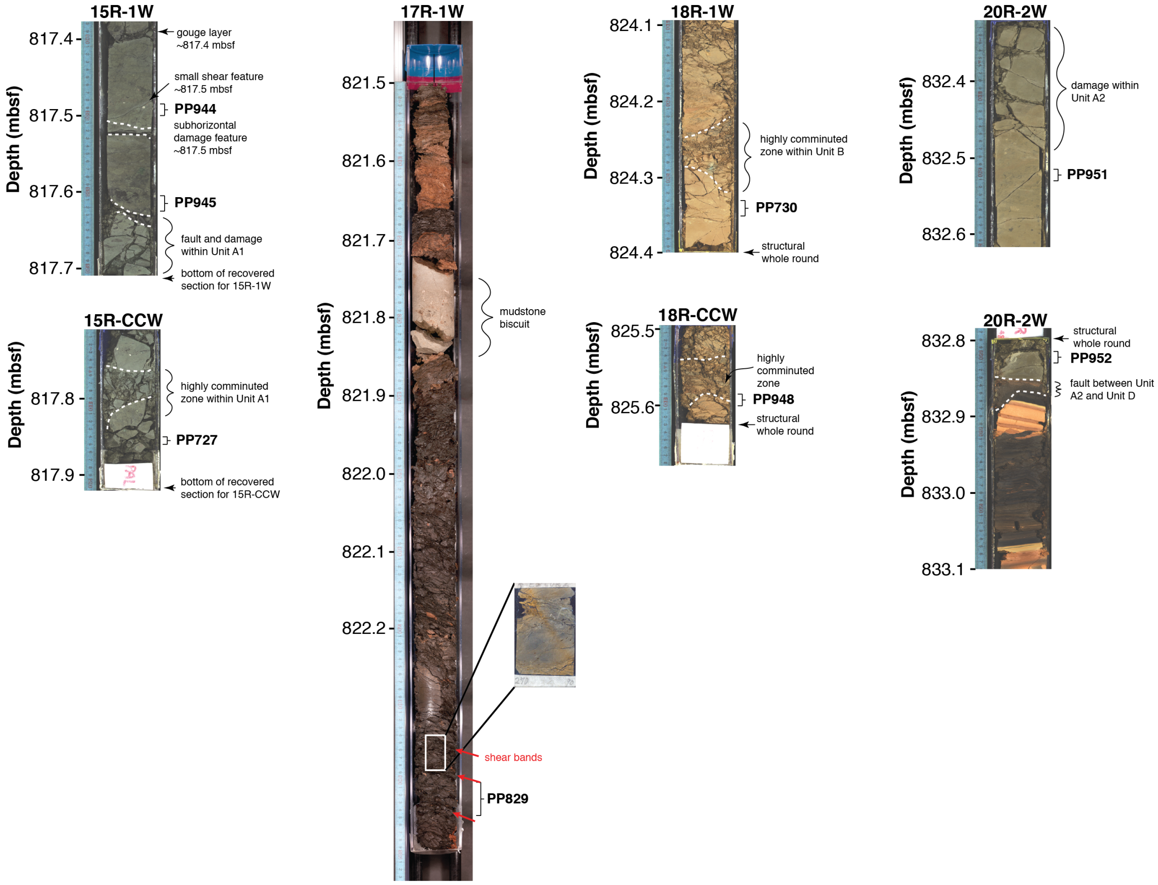

Supplementary Fig. 6. Images of structures near samples with biomarker anomalies

Temperature rise on faults modeled was constrained by core observations. Sample locations are indicated with brackets and sample numbers. Damage features described in the supplemental material, as well as the locations of structural whole rounds and core boundaries are indicated by dotted white lines as well as annotations to the right of the core pictures[1]. The images of Core 17 were taken before structural whole rounds were removed from the core.

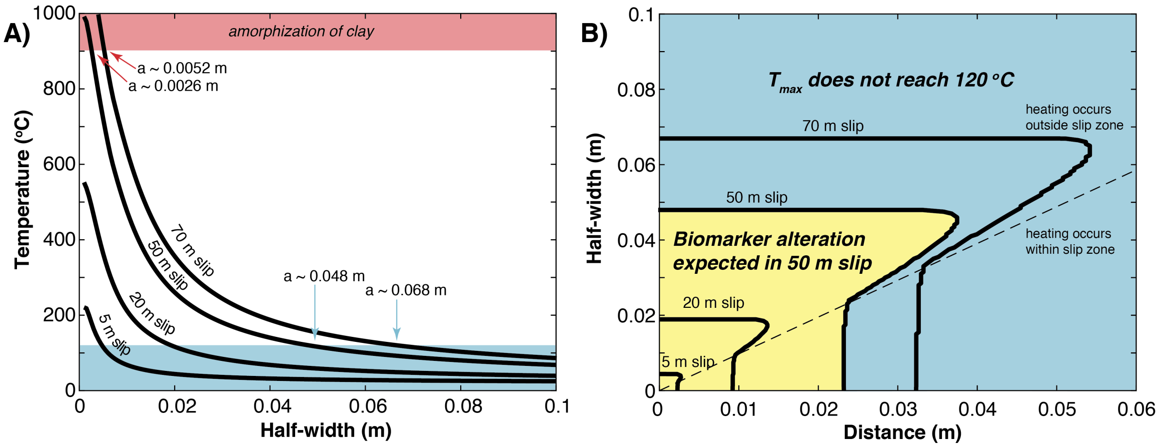

Supplementary Fig. 7. Fault heating modeling constraints

A) Temperature rise was additionally constrained by the fact that T_{max} could not exceed 900 ºC, at which point smectite should become amorphous[14,15] (red shaded region), or be less than 120 ºC, the minimum temperature for the thermal maturation of the biomarkers considered here (blue shaded region). These temperature bounds put limits on the maximum and minimum fault half-widths (*a*) that could be considered for a given amount of seismic slip. B) Yellow region shows acceptable half widths and distances from the faults where temperatures reach ≥120 ºC (i.e. biomarker reaction can occur).

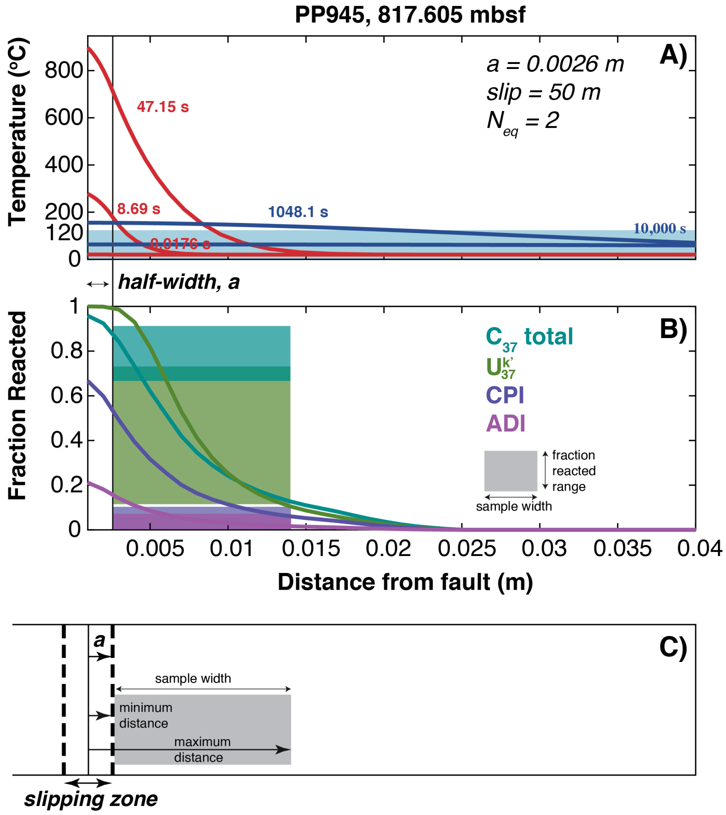

Supplementary Fig. 8. Example of a coupled fault heating and biomarker thermal maturity model for sample PP945

Example of a coupled fault heating and biomarker thermal maturity model for sample PP945 assuming a fault half-width of 0.0026 m and slip of 50 m for 2 earthquakes. Slip zone half-width is indicated by a black vertical line. (A) Temperature rise at a series of time-steps during (red) and after (blue) seismic slip at 1 m/s. The minimum temperature of biomarker reaction, 120 ºC, is indicated by the light blue bar. (B) Fractions reacted with distance from the fault calculated for C_{37} total, $U^{k'}_{37}$, CPI, and ADI are represented by colored curves. Corresponding sample measurements are indicated by translucent boxes in the appropriate color. The vertical extent of the boxes indicates the range of measured fractions reacted for each biomarker. The width of the boxes indicates the sample width. Note that because this sample was not within the candidate slip zone, only distances outside the slip zone are considered. (C) Schematic of model set-up. Candidate slipping zone is indicated by bold dashed lines. Half-width is half of the thickness of the candidate slipping zone. Grey box represents the sampled region of core with the minimum and maximum distances from the slipping zone indicated with arrows. This model fit is considered a success because all modeled biomarker fraction reacted values are within the range of measured biomarker fraction reacted values at an allowable distance from the fault structure. The probability that this sample can be fit by two 50 m slip events is determined by dividing the total number of successful model fits (considering the uncertainty in biomarker parameters) by the total number of models.

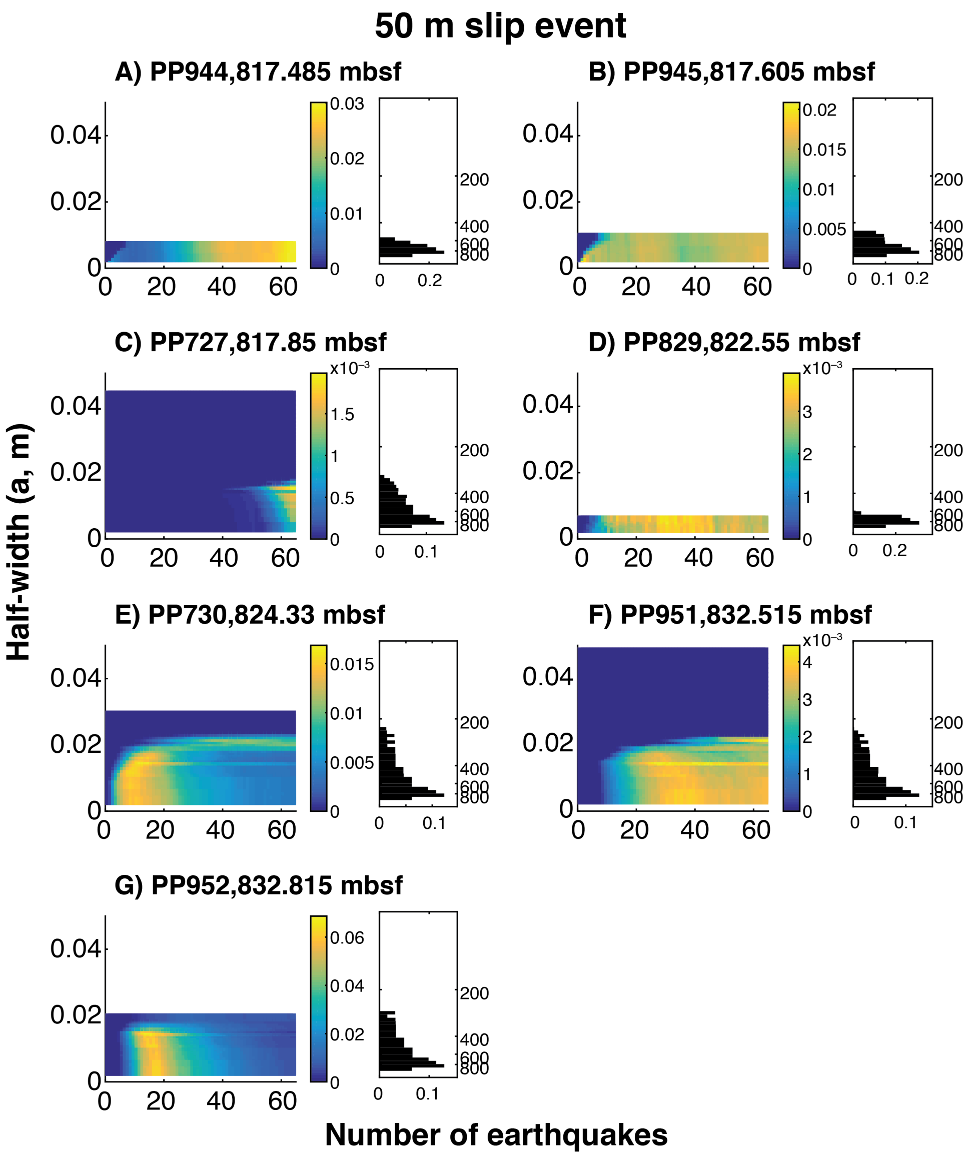

Supplementary Fig. 9. Results of coupled fault heating and biomarker reaction model for a range of fault half-width and number of earthquakes

(A–G) Results from coupled fault heating and biomarker reaction models, assuming 50 m of slip as observed in the Tohoku-Oki earthquake and 0.54 MPa coseismic shear stress as determined by Fulton et al. (2013). Colored plots show the probability of matching all biomarker constraints with a given fault half width, slipping in a given number of earthquakes. White areas correspond to half-widths that are either too thin (would yield a peak temperature above 900 ºC) or thicker than the observed fault structure recovered in the JFAST core. Histograms to the right of each colored plot show the probability of a match for a range of half-widths (summed across number of earthquakes) with the right-hand y-axis label showing the corresponding peak temperature.

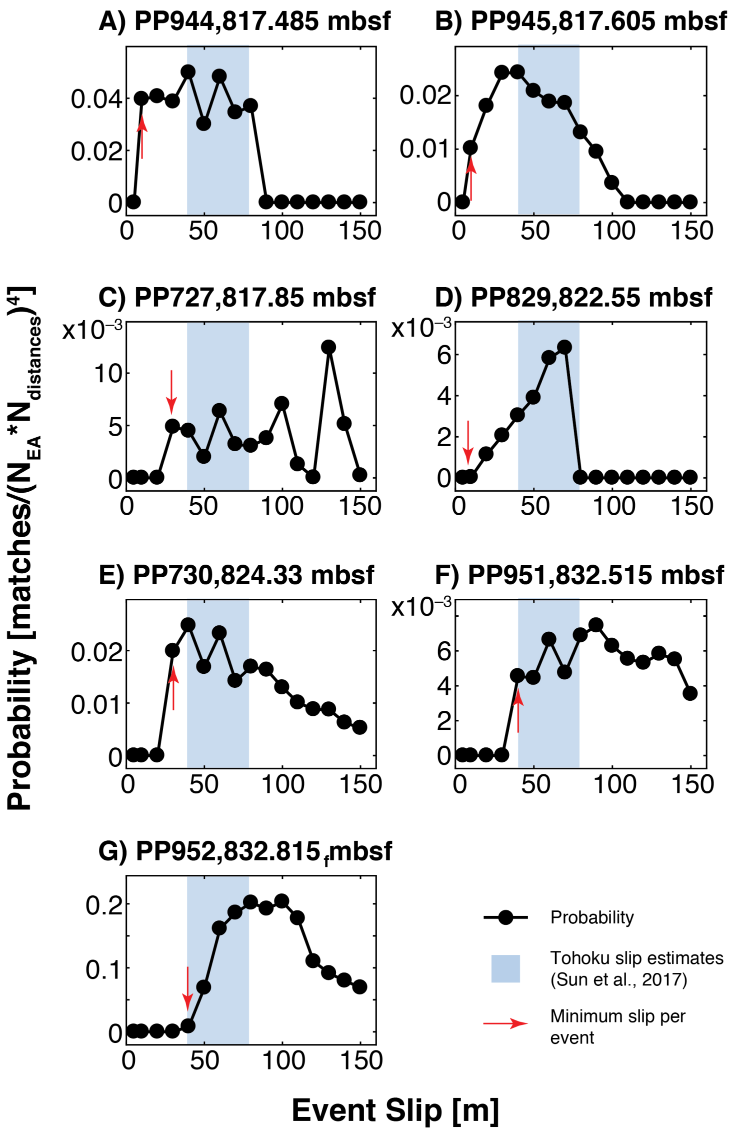

Supplementary Fig. 10. Probability that a given earthquake event slip explains observed biomarker anomalies

(A–G) Maximum probability (model matches to all four biomarker fraction reacted observations/ $(N_{EA}*W_{sample})^4$, where N_{EA} is the number of kinetic E and A rate pairs sampled from their joint uncertainty distribution and W_{sample} is the number of distances within the sample) for a range of slip magnitudes. Blue bars indicate the range of displacements that have been modeled for the Tohoku-Oki earthquake[13] and red arrows indicate the minimum required slip per event for each sample, corresponding to the minimum slip per event values plotted in Fig. 3. The lowest slip per event where the probability is greater than zero determines the minimum required slip to explain the biomarker measurements given their uncertainty and the uncertainty of the biomarker kinetics. Higher displacements are allowed but not required.

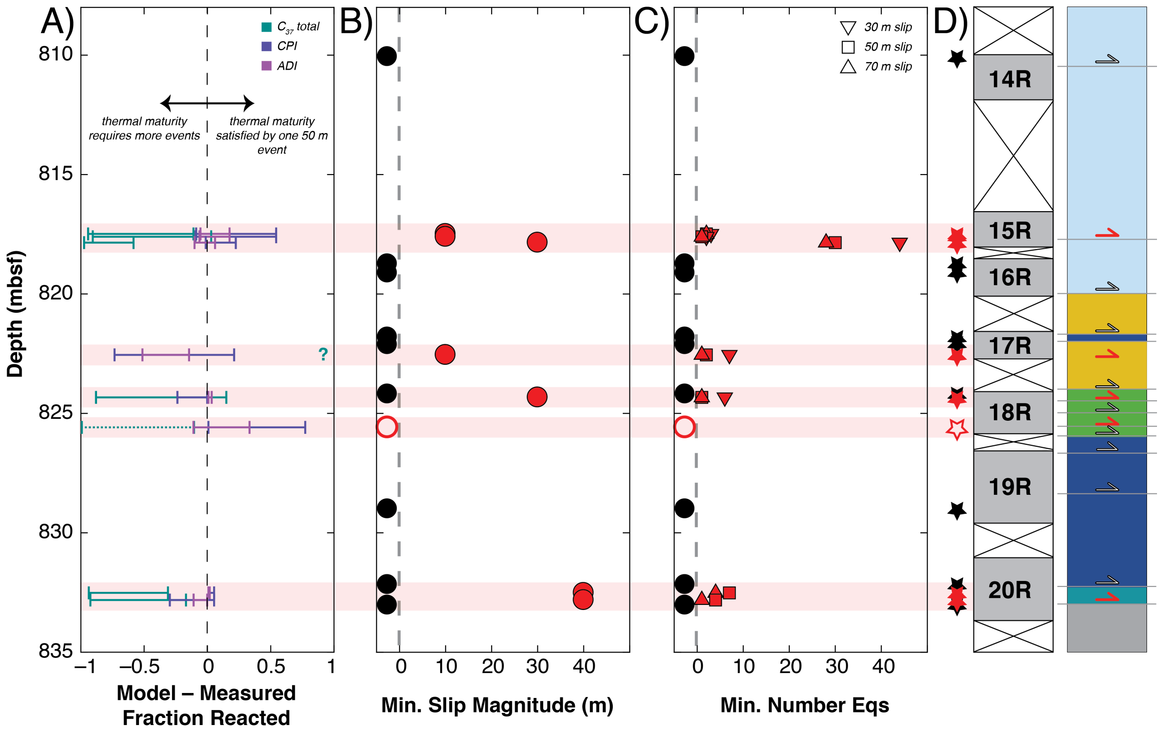

Supplementary Fig. 11. JFAST fault heating results

(A) Difference between modeled fraction reacted values for one Tohoku-Oki-sized earthquake with 50 m slip and measured biomarker values (C_{37} total, CPI, and ADI) indicates that all faults with heating anomalies could have hosted at least one event of this size. (B) Minimum slip magnitude and (C) minimum number of 30, 50, and 70 m slip events required to generate the observed biomarker anomalies in JFAST samples given the constraints discussed in the text. Red symbols in all plots indicate features with clear biomarker anomalies. Question mark in (A) indicates the pelagic clay sample, where alkenone concentrations are below the detection limit and cannot be used as a model constraint. Dashed line (A) and hollow symbol (D) represent sample PP948, which has alkenone concentrations below the quantification limit and is not modeled.

Supplementary Tables

Supplementary Table 1

Parameters for coupled fault heating biomarker reaction model.

Parameter	Value	Notes/Source
τ, shear stress	0.54 MPa	[12]
ρ_{fault}	1850 kg m^{-3}	Measured for JFAST at depth >650 mbsf[1]
c_p, specific heat capacity	$1.5157*10^3$ J kg^{-1} K^{-1}	Measured for JFAST[12]
α, thermal diffusivity	$3.92*10^{-7}$ m^2 s^{-1}	Measured for JFAST[12]
a, fault half-width	0.15 – 7 cm	varied in model
slip	5 – 150 m	varied in model
v, slip velocity	1 m s^{-1}	Wei et al. (2012)
t^*, earthquake duration	5 – 150 s	from slip/v
T_{bkg}, background temp.	20 °C	JFAST T measurements[12]
C_{37} $total$ E_a, activation energy	$3.60*10^4$ J mol^{-1}	best fit E_a[7]
C_{37} $total$ A, frequency factor	1.2 s^{-1}	best fit A[7]
$MK37{:}2$ E_a	$3.59*10^4$ J mol^{-1}	best fit E_a[7]
$MK37{:}2$ A	1.12 s^{-1}	best fit A[7]
$MK37{:}3$ E_a	$3.63*10^4$ J mol^{-1}	best fit E_a[7]
$MK37{:}3$ A	1.39 s^{-1}	best fit A[7]
CPI E_a	$3.38*10^4$ J mol^{-1}	best fit E_a[7]
CPI A	0.302 s^{-1}	best fit A[7]
ADI E_a	$3.23*10^4$ J mol^{-1}	best fit E_a[7]
ADI A	0.052 s^{-1}	best fit A[7]

Supplementary References

1. Chester, F. M., Mori, J., Eguchi, N., Toczko, S. & Scientists, E. 343/343T. *Volume 343-343T Expedition Reports Japan Trench Fast Drilling Project (JFAST).* (2012).

2. Rabinowitz, H. S., Savage, H. M., Plank, T., Kirkpatrick, J. D. & Rowe, C. D. Multiple major faults at the Japan Trench: Chemostratigraphy of the plate boundary at IODP Exp. 343: JFAST. *Earth Planet. Sci. Lett.* (2015).

3. Ujiie, K. *et al.* Low coseismic shear stress on the Tohoku-Oki megathrust determined from laboratory experiments. *Science* **342**, 1211–4 (2013).

4. Chester, F. M. *et al.* Structure and composition of the plate-boundary slip zone for the 2011 Tohoku-Oki earthquake. *Science* **342**, 1208–11 (2013).

5. Yang, T. *et al.* Strain decoupling across the décollement in the region of large slip during the 2011 Tohoku-Oki earthquake from anisotropy of magnetic susceptibility. *Earth Planet. Sci. Lett.* **381**, 31–38 (2013).

6. Herbert, T. D. *et al.* Late Miocene global cooling and the rise of modern ecosystems. *Nat. Geosci.* (2016). doi:10.1038/ngeo2813

7. Rabinowitz, H. S., Polissar, P. J. & Savage, H. M. Reaction kinetics of alkenone and n-alkane thermal alteration at seismic timescales. *Geochemistry, Geophys. Geosystems* **18**, (2017).

8. Kirkpatrick, J. D. *et al.* Structure and lithology of the Japan Trench subduction plate boundary fault. *Tectonics* **34**, (2015).

9. Yang, T., Dekkers, M. J. & Zhang, B. Seismic heating signatures in the Japan Trench subduction plate-boundary fault zone: evidence from a preliminary rock. *Geophys. J. Int.* **205**, 332–344 (2016).

10. Janssen, C. *et al.* Co-seismic and/or a-seismic microstructures of JFAST 343 core samples from the Japan Trench. *Mar. Geol.* **362**, 33–42 (2015).

11. Wenger, L. M. *et al.* Drill Bit Metamorphism: Recognition and Impact on Show Evaluation. in *SPE Annual Technical Conference and Exhibition* (2009). doi:10.2118/125218-MS

12. Fulton, P. M. *et al.* Low coseismic friction on the Tohoku-Oki fault determined from temperature measurements. *Science* **342**, 1214–7 (2013).

13. Sun, T., Fujiwara, T., Kodaira, S., He, J. & Wang, K. Large fault slip peaking at trench in the 2011 Tohoku-oki earthquake. *Nat. Commun.* **8**, 1–8 (2017).

14. Spray, J. G. A physical basis for the frictional melting of some rock-forming

minerals. *Tectonophysics* **204**, 205–221 (1992).

15. Noyan, H., Önal, M. & Sarikaya, Y. Thermal deformation thermodynamics of a smectite mineral. *J. Therm. Anal. Calorim.* **91**, 299–303 (2008).

16. Fulton, P. M. & Harris, R. N. Thermal considerations in inferring frictional heating from vitrinite reflectance and implications for shallow coseismic slip within the Nankai Subduction Zone. *Earth Planet. Sci. Lett.* **335–336**, 206–215 (2012).

17. Keren, T. T. & Kirkpatrick, J. D. The damage is done: Low fault friction recorded in the damage zone of the shallow Japan Trench decollement. *J. Geophys. Res.* 3804–3824 (2016). doi:10.1002/2015JB012311

18. Keren, T. T. & Kirkpatrick, J. D. *Data report : tectonic and induced structures in the JFAST core 1*. **343/343T**, (2016).

19. Sawai, M., Hirose, T. & Kameda, J. Frictional properties of incoming pelagic sediments at the Japan Trench: implications for large slip at a shallow plate boundary during the 2011 Tohoku earthquake. *Earth, Planets Sp.* **66**, 65 (2014).

20. Rice, J. R. Heating and weakening of faults during earthquake slip. *J. Geophys. Res.* **111**, 1–29 (2006).
