# Supplementary material for: Earthquake slip surfaces identified by biomarker thermal maturity within the 2011 Tohoku-Oki earthquake fault zone
Source: Nat Commun. 2020 Jan 27;11:533. doi: 10.1038/s41467-020-14447-1 (PMC6985169; doi:10.1038/s41467-020-14447-1)
Supplement: Supplementary file 3 — Description of Additional Supplementary Files [file 41467_2020_14447_MOESM3_ESM.pdf]

Description of Additional Supplementary Files

File Name: Supplementary Data 1.
Description: Alkenone, total organic carbon (TOC), and n-alkane concentrations measured in samples from the JFAST core and Site 436.

File Name: Supplementary Data 2.
Description: Biomarker indices (C37 total, Uk'37, CPI, and ADI) calculated for samples from the JFAST core and Site 436.

File Name: Supplementary Data 3.
Description: Model results from fault heating models run on JFAST samples showing clear biomarker heating signals. Minimum required slip magnitudes correspond to values shown by red arrows in Supplementary Figure 10. The minimum number of earthquakes corresponds to the minimum number of earthquakes of the stated slip magnitude required to match the biomarker observations in the JFAST samples. Reported minimum temperature estimates are taken from the distributions calculated for the minimum sized earthquake for each sample. Shear stress in these models is 0.54 MPa.
